# Clinicopathological Features of Extranodal Head and Neck Lymphomas

**DOI:** 10.3390/diagnostics16081168

**Published:** 2026-04-15

**Authors:** Füruzan Kacar Döger, Büşra Ekinci, Yeşim Başal

**Affiliations:** 1 Department of Pathology, Aydın Atatürk State Hospital, Aydın 09020, Turkey; 2 Department of Pathology, Aydın Adnan Menderes University, Aydın 09100, Turkey; busraekinci16@gmail.com; 3 Department of Otolaryngology, Aydın Adnan Menderes University, Aydın 09100, Turkey; yesimdurgun@gmail.com

**Keywords:** extranodal lymphoma, head and neck neoplasms, lymphoma, pathology

## Abstract

**Objective:** Primary extranodal lymphomas of the head and neck region are relatively rare and represent a biologically distinct subset. The diagnosis and differential diagnosis of head and neck lymphomas are important and deserve special attention. The aim of the present study was to retrospectively evaluate patients diagnosed with primary head and neck lymphomas at the Department of Pathology between January 2020 and January 2026. Histopathological subtypes, localization, relative frequencies, and overall survival were analyzed. **Materials and Methods:** This retrospective study included 31 cases diagnosed with lymphoma involving the head and neck region. Medical records were reviewed. Histopathological slides were re-evaluated under light microscopy by experienced pathologists. All cases were classified according to the current World Health Organization (WHO) classification of tumors of hematopoietic and lymphoid tissues. An extensive immunohistochemical panel was applied. Statistical analysis was performed using SPSS statistical software (version 27.0; IBM Corp., Armonk, NY, USA). **Results:** The study group included 31 patients with head and neck lymphoma. The most common histological type was diffuse large B-cell lymphoma (DLBCL) (54.8%). Other histological subtypes included follicular lymphoma (FL), mantle cell lymphoma (MCL), extranodal NK/T-cell lymphoma (NKTCL), anaplastic large cell lymphoma (ALCL), and Hodgkin lymphoma (HL). The most common location was the tonsil (38.7%). Other locations included the nasopharynx, oral cavity, nasal cavity, salivary glands, and thyroid. Epstein–Barr virus (EBV) positivity was detected in two patients (6.5%), and human immunodeficiency virus (HIV) infection was identified in two patients (6.5%). At the time of the last follow-up, 27 patients (87.1%) were alive, whereas four patients (12.9%) had died. The mortality rate was 12.9%. The median overall survival was 28 months (95% CI: 10–45). **Conclusions:** Malignant lymphoma should be considered when evaluating head and neck masses, and histopathological assessment of the affected tissue remains the cornerstone of diagnosis.

## 1. Introduction

Malignant lymphomas of lymphoid cell origin constitute approximately 5% of all malignant neoplasms arising in the head and neck region [1,2]. Among these, extranodal head and neck lymphomas represent a distinct clinical entity, as they predominantly originate in extranodal tissues rather than lymph nodes. Although the gastrointestinal tract is the most common site of extranodal lymphoma involvement, the head and neck region is the second most frequently affected anatomical location [1,2,3].

These lymphomas are initially diagnosed in the head and neck region and may present with or without involvement of contiguous regional lymph nodes at the time of diagnosis. A wide variety of anatomical sites can be affected, including Waldeyer’s ring, salivary glands, nasal cavity, paranasal sinuses, orbit, oral cavity, and larynx. Due to their diverse anatomical distribution and histopathological subtypes, extranodal head and neck lymphomas often exhibit heterogeneous clinical manifestations, radiological findings, and biological behavior, which may complicate early diagnosis and management [1,2,3,4].

According to the World Health Organization (WHO) classification, lymphomas are broadly categorized into Hodgkin lymphoma (HL) and non-Hodgkin lymphoma (NHL) [2,5]. The majority of lymphomas occurring in the head and neck region are non-Hodgkin lymphomas of B-cell origin, with diffuse large B-cell lymphoma (DLBCL) being the most common histopathological subtype [2,4,5]. In contrast, HL rarely presents as a primary extranodal disease in the head and neck region. It typically manifests as a localized nodal disease, most commonly involving the mediastinum and cervical lymph nodes, while primary extranodal presentation is uncommon [6].

Waldeyer’s ring constitutes the most commonly involved site, accounting for more than half of all reported cases of extranodal head and neck lymphomas [1,2,7]. Other frequently affected extranodal sites include the nasal cavity and paranasal sinuses, oral cavity, ocular adnexa, nasopharynx, and thyroid gland [2,8,9,10,11,12,13,14].

Primary extranodal lymphomas of the head and neck represent a heterogeneous group of malignancies, reflecting substantial geographic variations in epidemiology, clinical behavior, and etiological factors worldwide [1,2,9,13,15]. Furthermore, epidemiological studies have demonstrated a steady increase in the global incidence of lymphoma, with an estimated annual rise of approximately 3–4% [2,3].

Chemotherapy is the most frequently employed treatment approach, with radiotherapy and immunotherapy used as adjunctive modalities [7,13]. The presence of complex pathological features frequently complicates early clinical recognition, leading to delays in diagnosis and treatment. As a result, disease progression may occur, and patients with advanced-stage head and neck lymphomas tend to experience poor clinical outcomes, including decreased overall survival [7].

The aim of the present study was to retrospectively evaluate patients diagnosed with primary head and neck lymphomas at the Department of Pathology between 2020 and 2026. Histopathological subtypes, relative frequencies, and overall survival were systematically analyzed.

## 2. Materials and Methods

Clinical data from 31 patients diagnosed with head and neck lymphoma who were hospitalized at Aydın Adnan Menderes University Faculty of Medicine between January 2020 and January 2026 were retrospectively collected and analyzed. Only patients who received their initial diagnosis and first-line treatment at our institution were included in the study. The diagnosis of lymphoma was confirmed based on histopathological examination and immunohistochemical analysis of tissue samples. Patients with incomplete or insufficient medical records, those with a history of other malignant tumors, and cases with suboptimal or poor-quality paraffin-embedded tissue sections were excluded from the analysis.

Medical records were retrospectively reviewed to collect data on demographic characteristics, initial clinical presentation, and potential risk factors. When required, patients or their relatives were contacted to obtain follow-up information regarding clinical status and survival outcomes. All patients were evaluated for known risk factors, including human immunodeficiency virus (HIV), hepatitis C virus (HCV), hepatitis B virus (HBV), and Epstein–Barr virus (EBV). Imaging studies—such as Doppler ultrasonography, magnetic resonance imaging (MRI), and/or contrast-enhanced computed tomography (CT)—were performed to assess lesion extent and evaluate possible bone involvement. Histopathological slides corresponding to each case were retrieved from the pathology archives and independently re-evaluated under light microscopy by experienced pathologists. All cases were classified according to the World Health Organization (WHO, 5th ed.) classification of tumors of hematopoietic and lymphoid tissues [5] as follows: diffuse large B-cell lymphoma (DLBCL), follicular lymphoma (FL), mantle cell lymphoma (MCL), anaplastic large cell lymphoma (ALCL), NK/T-cell lymphoma (NKTCL), and Hodgkin lymphoma (HL).

In the 5th edition of the WHO classification, DLBCL is categorized into germinal center B-cell-like (GCB) and non-germinal center B-cell–like (non-GCB) subtypes based on immunohistochemical findings. Follicular lymphoma (FL) grading is determined by counting the number of centroblasts per high-power field (HPF) in 10 representative neoplastic follicles: grade 1 is defined as 0–5 centroblasts per HPF, grade 2 as 6–15 centroblasts per HPF, and grade 3 as >15 centroblasts per HPF [5].

Immunohistochemical (IHC) analyses were performed using a fully automated immunostaining system on formalin-fixed, paraffin-embedded tissue sections. An extensive immunohistochemical panel was applied, including EMA, CD2, CD3, CD5, CD10, CD20, CD23, CD45, MUM-1, CD56, CD79a, BCL-2, BCL-6, TIA-1, granzyme B, CD30, and CD15. Additional IHC stains were performed in selected cases when required. The complete immunohistochemical panel is summarized in Table 1.

IHC positivity for BCL-2 was defined using a cutoff value of ≥70%, while positive expression of CD10, BCL-6, and MUM-1 was determined using a threshold of ≥30% [16,17]. Based on Hans’ classification system, patients were stratified into GCB and non-GCB subtypes [18]. Disease staging was performed for all patients in accordance with the Ann Arbor staging system, which was initially developed in 1971 for HL and later adopted for the staging of NHL [19]. Furthermore, patients were categorized into two groups (A and B) according to the absence or presence of systemic B symptoms. Systemic B symptoms were defined as unexplained weight loss exceeding 10% of body weight within a six-month period, unexplained fever, and/or night sweats.

Each patient received treatment with chemotherapy, immunotherapy, and/or radiotherapy (RT).

### 2.1. Statistical Analysis

Statistical analyses were performed using IBM SPSS Statistics (version 27.0; IBM Corp., Armonk, NY, USA). Continuous variables were summarized using appropriate descriptive statistics, including the mean and standard deviation (SD) for normally distributed data, and the median with interquartile range (IQR) for non-normally distributed data. Categorical variables were expressed as frequencies and percentages. For comparisons of categorical variables, the chi-square test or Fisher’s exact test was used, as appropriate. Appropriate parametric or non-parametric tests were applied for continuous variables. A *p*-value < 0.05 was considered statistically significant. Survival analysis was performed using the Kaplan–Meier method with the log-rank test.

### 2.2. Ethics Statement

This retrospective study was reviewed and approved by the Institutional Ethics Committee of Aydın Adnan Menderes University (Approval No: E-53043469-050.04-794560; date: 23 January 2026). The study was conducted using archived clinical and pathological materials. Due to the retrospective nature of the study and the use of existing data, the requirement for informed consent was waived by the ethics committee. All patient data were fully anonymized prior to analysis and were evaluated in strict accordance with the ethical principles outlined in the Declaration of Helsinki.

The authors declare that this study did not receive any financial support from public, commercial, or non-profit funding agencies and that there are no conflicts of interest related to this work.

## 3. Results

A total of 31 patients were included in the study. Of these, 19 patients (61.3%) were male and 12 (38.7%) were female. Male to female ratio was 1.58:1. The mean age at diagnosis was 60.9 ± 18.4 years (IQR: 13–87 years), with a median age of 69.7 years.

In addition, 27 cases (87.1%) were at stage I or II, while 4 cases (12.9%) were at stage III or IV according to the Ann Arbor staging system.

The clinical manifestations of head and neck lymphoma were diverse, reflecting differences in primary sites, pathological subtypes, and symptom severity. Statistical analysis showed that 10 patients (32.3%) presented with B symptoms, including unexplained weight loss, fever, and/or night sweats. Primary site swelling was observed in 5 cases (16.1%). Pharyngeal pain accompanied by dysphagia occurred in 4 patients (12.9%). Dyspnea (1 case, 3.2%) and hoarseness (2 cases, 6.5%) were less common. Nasal symptoms included obstruction (3 cases, 9.7%), rhinorrhea (2 cases, 6.5%), nasal odor (2 cases, 6.5%), and epistaxis (1 case, 3.2%).

In all 31 patients diagnosed with head and neck lymphoma, pathological specimens were obtained either through surgical procedures or via endoscopic biopsy using flexible nasolaryngoscopy.

Physical examination and endoscopic findings revealed that the most common morphological presentation was a mass (5 cases, 16.1%), followed by localized ulceration (4 cases, 12.9%), a tumor with associated ulceration (3 cases, 9.7%), and isolated mucosal swelling (2 cases, 6.5%). Fisher’s exact test was used to examine the relationship between morphological features and pathological subtypes. No statistically significant differences were observed (*p* > 0.05).

EBV positivity was detected in two patients (6.5%), and HIV infection was identified in two patients (6.5%). Among the EBV-positive cases, one was diagnosed as DLBCL localized to the tonsil, whereas the other was NKTCL involving the nasal cavity. Among the HIV-positive cases, one consisted of NKTCL localized to the oral mucosa, while the other was DLBCL involving the oral cavity.

**Figure 1:**
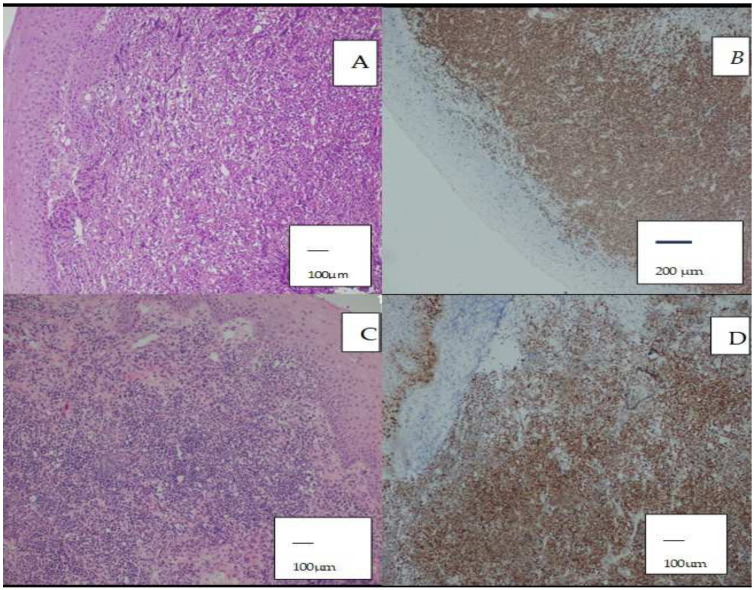
(**A**) Diffuse large B-cell lymphoma (DLBCL) in the tonsil (H&E, ×100; scale bar = 100 µm). (**B**) CD20 positivity in DLBCL (×40; scale bar = 200 µm). (**C**) Mantle cell lymphoma (MCL) in the tonsil (H&E, ×100; scale bar = 100 µm). (**D**) Cyclin D1 positivity in MCL (×100; scale bar = 100 µm). Brown staining indicates positive immunohistochemical expression, while blue represents nuclear counterstaining (hematoxylin).

**Figure 2:**
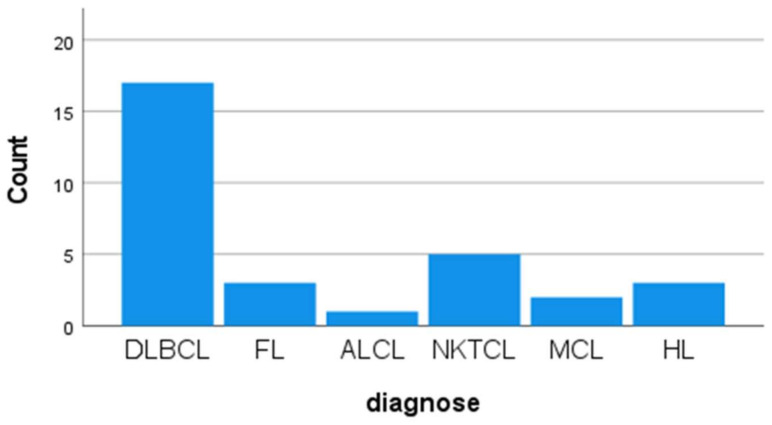
Histopathological distribution of lymphoma subtypes. Diffuse large B cell lymphoma (DLBCL), Follicular Lymphoma (FL), Anaplastic large cell lymphoma (ALCL), N/K T cell lymphoma (NKTCL), Mantle cell lymphoma (MCL), Hodgkin Lymphoma (HL).

Cases were classified according to histopathological subtype. The most common subtype was DLBCL (Figure 1A,B), accounting for 17 cases (54.8%). Other subtypes included FL in three cases (9.7%), MCL in two cases (6.5%) (Figure 1C,D), NKTCL in five cases (16.1%), ALCL in one case (3.2%), and HL in three cases (9.7%). The distribution of histopathological subtypes is shown in Figure 2.

Twelve lymphomas were located in the tonsils (38.7%), eight in the nasopharynx (25.8%), five in the oral mucosa (16.1%), three in the nasal cavity (9.7%), two in the salivary glands (6.5%), and one in the thyroid gland (3.2%). The specific relationship between pathological types and primary sites is shown in Table 2.

According to the immunohistochemical results, the 31 cases were divided into 23 (74.2%) with Ki-67 < 50% and 8 (25.8%) with Ki-67 ≥ 50. There was no statistically significant difference in survival between patients with high versus low Ki-67 expression (*p* > 0.05).

**Figure 3:**
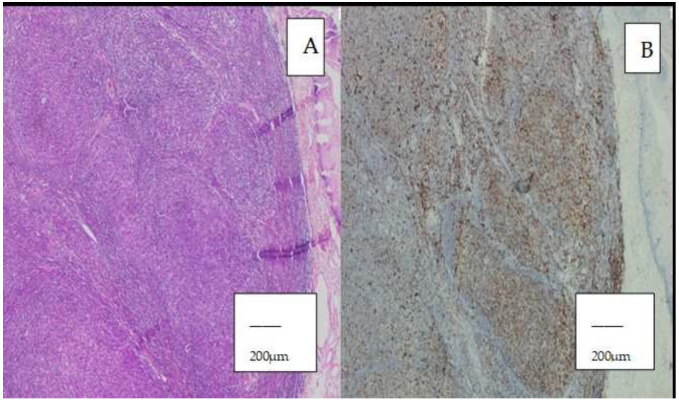
(**A**) Follicular lymphoma (FL) (H&E, ×40; scale bar = 200 µm). (**B**) BCL2 positivity in FL (×40; scale bar = 200 µm). Positive staining is indicated by brown coloration.

Bcl-2 positivity was observed in 18 cases (58.1%) (Figure 3A,B), whereas 13 cases (41.9%) were negative.

All laboratory and imaging examinations were completed and analyzed for all the 31 patients before treatment.

**Figure 4:**
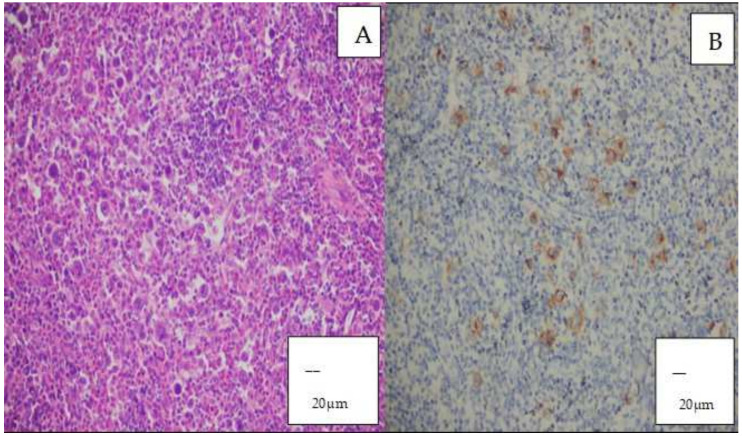
(**A**) Hodgkin lymphoma (HL) (H&E, ×200; scale bar = 20 µm). (**B**) Hodgkin–Reed–Sternberg cells showing positive staining for CD30 (×200; scale bar = 20 µm). Positive staining is indicated by brown coloration.

All cases of Hodgkin lymphoma identified in this study were histopathologically classified as the mixed cellularity subtype. In these cases, Reed–Sternberg cells or Reed–Sternberg-like cells were observed in a background rich in lymphocytes, eosinophils, plasma cells, and histiocytes, and demonstrated positive staining for CD30 on immunohistochemical analysis (Figure 4A,B).

Among all 31 patients, 17 had pathological DLBCL, of which 14 (82.4%) were classified as the GCB subtype and 3 (17.6%) as the non-GCB subtype.

Treatment of head and neck lymphomas was tailored according to histologic subtype, disease stage, and patient factors. Aggressive B-cell lymphomas (e.g., DLBCL) were treated with R-CHOP ± radiotherapy. Follicular lymphoma was managed with watch-and-wait, radiotherapy, or rituximab-based chemotherapy for advanced cases. Mantle cell lymphoma was treated with chemotherapy, sometimes followed by autologous stem cell transplantation. T-cell and NK/T-cell lymphomas were treated with CHOP-like regimens ± radiotherapy, while Hodgkin lymphoma was managed with ABVD ± radiotherapy. All patients were referred to oncologists to receive appropriate therapy.

**Figure 5:**
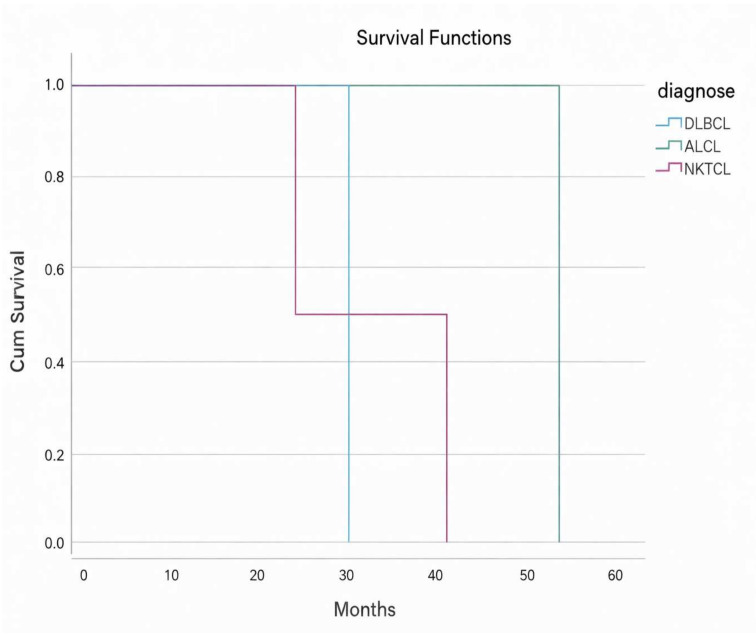
Kaplan–Meier survival curves showing cumulative survival probabilities according to diagnosis group DLBCL, ALCL, and NKTCL. No statistically significant difference in survival was observed between groups (log-rank test, *p* > 0.05). Kaplan–Meier survival curves are shown for cases with observed deaths; MCL and FL cases are not represented in the curves due to the absence of death events. Abbreviations: diffuse large B cell lymphoma (DLBCL), anaplastic large cell lymphoma (ALCL), NK T-cell lymphoma: (NKTCL).

**Figure 6:**
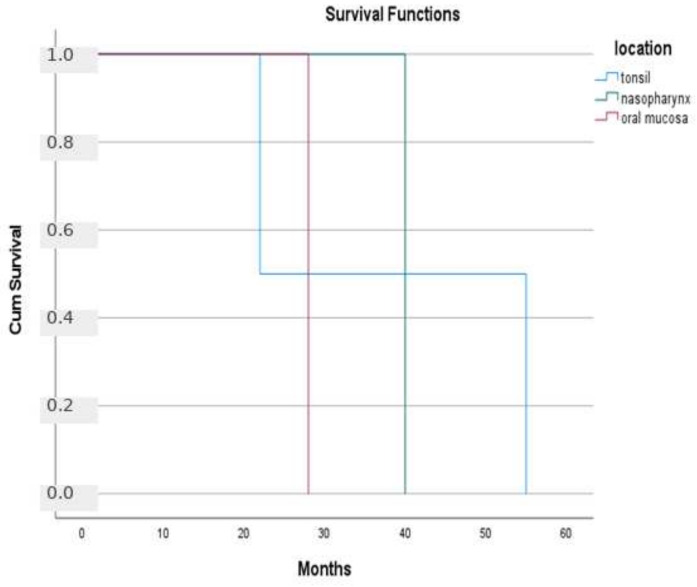
Comparison of survival distributions according to tumor location using the log-rank test. No statistically significant difference was observed between the groups (*p* > 0.05). Kaplan–Meier curves are not shown for the nasal cavity, salivary gland, and thyroid cases, as no deaths occurred in these groups.

At the time of the last follow-up, 27 patients (87.1%) were alive, whereas four patients (12.9%) had died, resulting in a mortality rate of 12.9%. The median overall survival was 28 months (95% CI: 10–45). A log-rank test was performed to compare survival distributions across the diagnostic groups, and no statistically significant differences were observed (*p* > 0.05) (Figure 5). Similarly, when survival was analyzed according to tumor location (tonsil, nasopharynx, and oral mucosa), no significant impact on survival was found (*p* > 0.05) (Figure 6). Overall, these findings suggest that neither the diagnostic group nor tumor location had a statistically significant effect on survival in this cohort.

**Figure 7:**
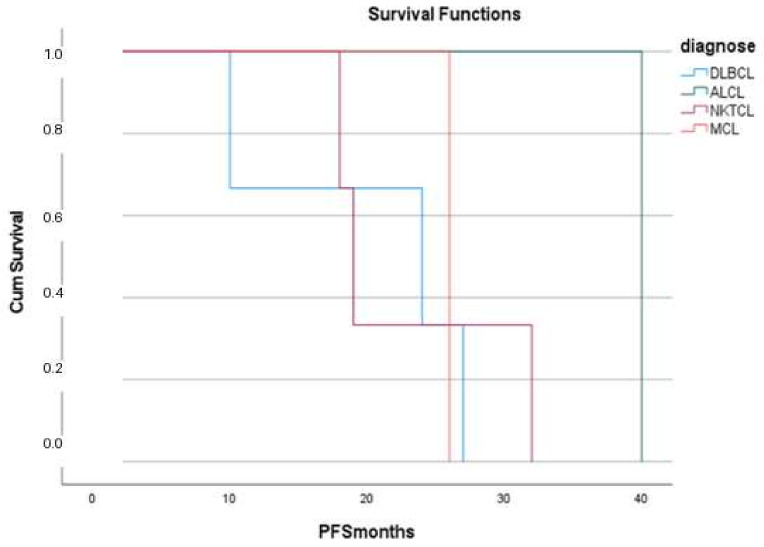
Comparison of progression-free survival (PFS) according to lymphoma type using the log-rank test. No statistically significant differences were observed between the groups (*p* > 0.05). Abbreviations: diffuse large B cell lymphoma (DLBCL), mantle cell lymphoma (MCL), anaplastic large cell lymphoma (ALCL), NK T-cell lymphoma: (NKTCL).

Disease progression was observed in 8 cases (25.8%), while 23 cases (74.2%) remained progression-free. No significant differences in disease progression were observed among lymphoma subtypes (*p* > 0.05) (Figure 7).

Kaplan–Meier survival analyses were performed for the cohort. No statistically significant differences were observed between lymphoma subtypes or tumor sites. Due to the small sample size and heterogeneity of the cases, all analyses are presented for exploratory purposes only and should not be interpreted as evidence of prognostic differences.

## 4. Discussion

In the present study, a retrospective evaluation of 31 patients with extranodal lymphoma of the head and neck region was conducted. Consistent with previous reports, lymphomas in this anatomical area demonstrate highly heterogeneous and nonspecific clinical presentations, often mimicking benign, inflammatory, or infectious conditions [2,3,8,9]. Moreover, the complex anatomy of the head and neck, combined with the diverse histopathological spectrum of extranodal lymphomas, substantially complicates early and accurate diagnosis. These diagnostic challenges may lead to delayed recognition and postponement of appropriate therapeutic interventions, potentially adversely affecting treatment outcomes and contributing to more advanced disease at presentation, ultimately impacting prognosis [2,3].

Previous studies have reported that lymphomas of the head and neck region are more common in men than in women [7,20,21,22]. In our study, a slight male predominance was observed (M/F: 1.58:1). Gender distribution did not differ significantly from that reported in the literature [1,2,3,4].

Regarding prognostic factors, age, disease stage, and general health status are of critical importance. Several studies have shown that patients older than 60 years, those with advanced-stage disease, and those with poor overall health have higher mortality rates [3,4]. In our cohort, the mean age was 60.9 ± 18.4 years (range: 13–87 years), with a median age of 69.7 years. Twenty-one patients (67.7%) were older than 60 years, while 10 patients (32.3%) were younger. A statistically significant difference in mortality was observed between these two groups (*p* = 0.002). Consistent with previous reports, our findings indicate that age at diagnosis is a significant prognostic factor.

In line with earlier studies [2,3,4,8,9,13], lymphomas in the head and neck region in our cohort exhibited highly heterogeneous and nonspecific clinical features, making early diagnosis challenging. These presentations may closely resemble benign inflammatory conditions such as pharyngitis, sinusitis, and tonsillitis, as well as other head and neck malignancies and various otorhinolaryngological disorders.

The treatment of malignant lymphoma is highly variable and depends on an accurate diagnosis [7,8,19,20]. In our cohort, 28 cases were non-Hodgkin lymphoma (NHL) and 3 were Hodgkin lymphoma (HL). Among NHL cases, diffuse large B-cell lymphoma (DLBCL) was the most common subtype, accounting for 54.8% of cases, which is consistent with published data [1,2,3,4,9,10,11,12,13,17]. Compared with Wei et al. [9], our findings demonstrate similar patterns, with DLBCL as the predominant subtype and a slight male predominance. The tonsil was also the most frequently involved site in both cohorts. However, our study included a higher proportion of NK/T-cell lymphoma (NKTCL) cases (16.1%), possibly reflecting regional, ethnic, or immunodeficiency-related differences. In terms of survival, our older cohort (median age 69.7 years) had a median overall survival (OS) of 28 months, with only four deaths recorded, limiting robust comparisons. In contrast, Wei MG et al. [9] analyzed a larger adolescent and young adult population. Both studies highlight the challenges posed by heterogeneous clinical presentations and emphasize the importance of accurate histopathological and immunohistochemical evaluation.

Approximately half of extranodal lymphomas in the head and neck region occur in Waldeyer’s ring, with the tonsils being the most commonly involved site [1,2,3,4]. In our study, the tonsil was the most frequent location (38.7%), followed by the nasopharynx (25.8%). Fisher’s analysis revealed no statistically significant association between tumor location and pathological subtype (*p* > 0.05).

Nasal cavity lymphomas are reported to be more prevalent among individuals of Asian and Latin American descent than among those of European ancestry, suggesting a role for genetic or ethnic susceptibility factors. This predisposition appears to be further enhanced by Epstein–Barr virus (EBV) infection, which is strongly associated with certain lymphoma subtypes, particularly NKTCL, nasal type [1,8,21]. In our series, three cases originated in the sinonasal region: two were NKTCL and one was DLBCL. EBV positivity was detected in one case, supporting the established association. Additionally, one patient in this subgroup was HIV-positive, a condition associated with immune dysregulation and increased susceptibility to aggressive lymphoid malignancies. These findings underscore the importance of considering viral infections and immunosuppressive states in diagnosis and risk stratification [20,21].

Primary lymphomas of the salivary glands are rare and present a diagnostic challenge. The parotid gland is the most frequently affected site, and mucosa-associated lymphoid tissue (MALT) lymphoma is generally the most common subtype, often associated with chronic inflammatory or autoimmune conditions [1,2,22]. In our study, however, no cases of MALT lymphoma were identified; instead, two parotid gland lymphomas were classified as DLBCL. This finding may reflect the small sample size or suggest possible transformation from an indolent lymphoma to a more aggressive subtype.

Primary oral lymphomas are uncommon. Oral NHL has been reported more frequently in patients with AIDS, and distinguishing infection-related lymphoproliferative disorders from lymphoma can be challenging [1,21,23]. EBV-positive mucocutaneous ulcers, particularly in immunocompromised patients, may mimic DLBCL, NKTCL, or even HL [1,19,23]. Accurate diagnosis requires the integration of histopathological, immunohistochemical, and clinical findings [19,20]. In our study, five cases involved the oral mucosa. One HIV-positive patient presented with an irregular gingival mass and was diagnosed with NKTCL; the diagnosis was straightforward due to its immunohistochemical profile.

Primary thyroid lymphoma (PTL) is an extremely rare malignancy, typically presenting as a rapidly enlarging neck mass causing compressive symptoms. It occurs predominantly in women and is strongly associated with Hashimoto’s thyroiditis, which significantly increases the risk [12,23,24]. Histologically, most thyroid lymphomas are DLBCL or extranodal MALT lymphoma. In our series, one female patient with a history of Hashimoto’s thyroiditis was diagnosed with DLBCL and presented with dyspnea.

The predominance of DLBCL in our study is consistent with the literature [1,2,9,10,13,15]. Reports of follicular lymphoma (FL) in the head and neck region are limited [25,26]. In our series, three cases (9.7%) were diagnosed as low-grade FL.

Mantle cell lymphoma (MCL) involving the tonsil is rare [14,27,28]; we identified two such cases (6.5%), both showing classical morphology and cyclin D1 positivity.

Anaplastic large cell lymphoma (ALCL) in the head and neck region is uncommon [29]. In our case, immunohistochemical analysis showed strong CD30 expression (>70%) and a high proliferative index (Ki-67), along with positivity for anaplastic lymphoma kinase (ALK).

T-cell lymphomas (TCLs) are rare and aggressive. When primary to the head and neck, they often present as extranodal NKTCL, nasal type [29,30,31]. We identified five NKTCL cases (16.1%), a higher proportion than typically reported in Western populations. This may reflect the small sample size, cohort characteristics, or increasing prevalence of immunodeficiency.

Classical Hodgkin lymphoma (HL) typically involves lymph nodes and rarely presents in the head and neck region [6,32,33]. In our study, three cases (9.7%) were identified, all of the mixed cellularity subtype. All patients had favorable outcomes and remained disease-free following therapy, consistent with the generally good prognosis of HL when diagnosed at an early stage.

Patients with advanced disease (Ann Arbor stage III–IV) had poorer outcomes, consistent with previous reports [7]. In our cohort, 27 patients (87.1%) were stage I–II, and 4 (12.9%) were stage III–IV. A statistically significant difference in mortality was observed between early and advanced stages (*p* = 0.018), although the number of events was limited.

The global incidence of lymphoma has been increasing by approximately 3–4% annually. Potential contributing factors include immunosuppression (e.g., AIDS), immunosuppressive therapies, infections, autoimmune diseases, and environmental influences [14,15,19,21].

In our study, only four deaths were recorded, while the remaining patients were alive. The median overall survival was 28 months (95% CI: 10–45) based on Kaplan–Meier analysis.

The lack of a statistically significant association between survival and tumor location or subtype may be attributed to the limited sample size and relatively short follow-up period. A small cohort reduces statistical power, making it difficult to detect true differences between groups. Additionally, insufficient follow-up time may prevent survival differences from becoming apparent. Therefore, these findings should be interpreted with caution, and larger studies with longer follow-up are needed to better evaluate the impact of tumor location on survival outcomes.

### Study Limitations

This study is limited by its retrospective design, incomplete hospital records in some cases, small sample size, and relatively short follow-up period, all of which may affect the generalizability of the findings.

In addition, molecular and cytogenetic analyses were not available for this cohort. Specifically, MYC immunohistochemistry and fluorescence in situ hybridization (FISH) analyses for MYC, BCL2, and BCL6 rearrangements were not performed. The absence of these data represents a limitation, particularly in the comprehensive characterization and risk stratification of diffuse large B-cell lymphoma cases according to current diagnostic standards.

Although a detailed immunohistochemical panel was applied, the lack of advanced molecular testing may have limited the full biological subclassification of aggressive lymphomas.

Furthermore, heterogeneity in treatment approaches among patients may have influenced survival outcomes.

## 5. Conclusions

Lymphomas are the most common non-epithelial tumors of the head and neck region. Therefore, accurate diagnosis and differential diagnosis are essential, and timely management remains crucial for improving outcomes in patients with extranodal head and neck lymphomas, given their heterogeneous and sometimes aggressive clinical presentation.

## Figures and Tables

**Table 1:** Immunohistochemical (IHC) panel used in the study.

Antijen	Clone	Source	Dilution
ALK	ALK1	DAKO, Glostrup, Demark	RTU
BCL-2	124	DAKO, Glostrup, Demark	RTU
BCL-6	PG-B6p	DAKO, Glostrup, Demark	RTU
CD3	M7254	DAKO, Glostrup, Demark	RTU
CD5	4C7	DAKO, Glostrup, Demark	RTU
CD8	C8/144B	DAKO, Glostrup, Demark	RTU
CD10	56C6	DAKO, Glostrup, Demark	RTU
CD20	L26	DAKO, Glostrup, Demark	1/200
CD30	Ber-H2	DAKO, Glostrup, Demark	RTU
CD43	DF-T1	DAKO, Glostrup, Demark	RTU
CD45	2B11 + PD7/26	DAKO, Glostrup, Demark	RTU
CD56	123C3	DAKO, Glostrup, Demark	RTU
CD79a	JCB117	DAKO, Glostrup, Demark	RTU
CYCLİN D1	EP12	DAKO, Glostrup, Demark	RTU
EMA	E29	DAKO, Glostrup, Demark	1/200
TİA1	TİA1	DAKO, Glostrup, Demark	RTU
Granzyme B	GrB-7	DAKO, Glostrup, Demark	RTU
CD15	M3631	DAKO, Glostrup, Demark	1/100
MUM1	MUM1p	DAKO, Glostrup, Demark	RTU

Abbreviations: IHC, immunohistochemistry; RTU, ready-to-use.

**Table 2:** The relationship between pathological types and primary sites.

	Tonsil	Naso-Pharynx	Oral Cavity	Nasal Cavity	Salivary Gland	Thyroid	Total
DLBCL	6	3	4	1	2	1	17
FL	2	1	0	0	0	0	3
ALCL	1	0	0	0	0	0	1
NKTCL	1	1	1	2	0	0	5
MCL	2	0	0	0	0	0	2
HL	0	3	0	0	0	0	3
Total	12	8	5	3	2	1	31

Abbreviations: diffuse large B cell lymphoma (DLBCL), Follicular lymphoma (FL), mantle cell lymphoma (MCL), anaplastic large cell lymphoma (ALCL) NK/ T-cell lymphoma: (NKTCL), Hodgkin lymphoma (HL).

## Data Availability

The data that support the findings of this study are not publicly available due to ethical and patient confidentiality restrictions.
